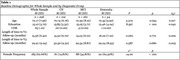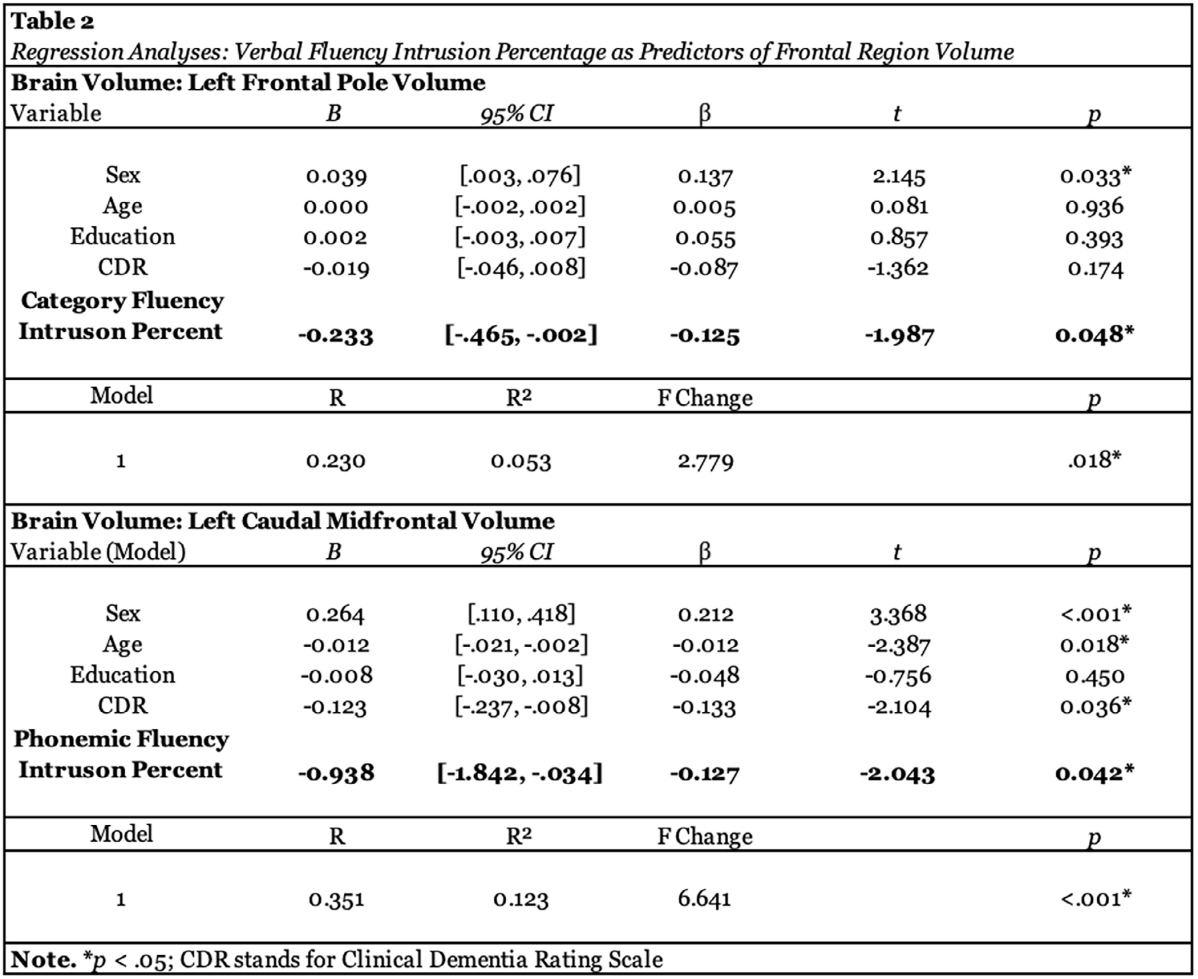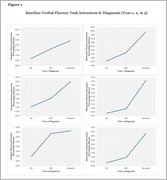# Intrusions in Verbal Fluency Tasks and Brain Volume in Mild Cognitive Impairment and Dementia: A Longitudinal Analysis

**DOI:** 10.1002/alz70857_107540

**Published:** 2025-12-26

**Authors:** Layaly Shihadeh, Stephen A. Coombes, Emily Ahne, Ranjan Duara, Glenn E. Smith, Idaly Velez‐Uribe, Warren W Barker, Monica Rosselli

**Affiliations:** ^1^ Florida Atlantic University, Davie, FL, USA; ^2^ 1Florida Alzheimer's Disease Research Center, Miami, FL, USA; ^3^ University of Florida, Gainesville, FL, USA; ^4^ Wien Center for Alzheimer's Disease and Memory Disorders, Miami Beach, FL, USA; ^5^ Mount Sinai Medical Center, Miami Beach, FL, USA

## Abstract

**Background:**

While intrusions in verbal memory tests are known to indicate a cognitive decline in aging, their role in verbal fluency (VF) tasks remains underexplored. Aims: to determine if intrusion percentages in phonemic (PF) and category (CF) VF tasks differ across cognitive diagnoses, longitudinally (across 3 years), and if they are associated with frontal lobe, temporal lobe, and hippocampal volumes.

**Methods:**

Participants were from the 1Florida Alzheimer's Disease Research Center (ADRC), including 102 CN, 160 MCI, and 34 dementia participants at year 1 (*M* (age) = 72.17; 62.66% female; Table 1), with year 2 and year 3 follow‐ups. The Clinical Dementia Rating (CDR‐GS) global score was used to determine cognitive status, (i.e. normal [CDR‐GS = 0], MCI [CDR‐GS = 0.5], or dementia [CDR‐GS = 1 ≥1]). Intrusion percentages from PF and CF tasks (collected every 15.36 months, on average) were analyzed alongside brain volumes (one‐time MRI scans collected at baseline).

**Results:**

Two separate MANCOVAs revealed intrusion percentages on VF tasks at baseline (year 1) significantly differed across year 2 diagnostic groups, as well as year 3 diagnostic groups (Figure 1), after controlling for demographic variables. This indicated intrusion percentages were significantly associated with diagnostic outcomes at year 2 and at year 3. Individuals with dementia exhibited higher intrusion percentages than cognitively normal individuals. Negative correlations were found between baseline VF intrusion percentages and brain volumes in frontal (left frontal pole and CF: *r* (296) = ‐.161, *p* = .005; left rostral midfrontal & CF: *r* (296) = ‐.151, *p* = .009; left caudal midfrontal & PF: *r* (285) = ‐.151, *p* = .010; right frontal pole & CF: *r* (296) = ‐.159, *p* = .006) and temporal regions (left inferior & CF: *r* (296) = ‐.158, *p* = .006: right middle and PF: *r* (296) = ‐.157, *p* = .008), after Bonferroni corrections. Regression analyses revealed higher baseline VF intrusion percentages were significantly associated with reduced left frontal pole and left caudal mid‐frontal volumes (Table 2).

**Conclusion:**

Intrusion percentages in VF tasks may be a sensitive marker of cognitive decline in aging and structural changes in the left frontal lobe.